# Auranofin Inhibits the Enzyme Activity of *Pasteurella multocida* Toxin PMT in Human Cells and Protects Cells from Intoxication

**DOI:** 10.3390/toxins9010032

**Published:** 2017-01-13

**Authors:** Stefan Carle, Thorsten Brink, Joachim H. C. Orth, Klaus Aktories, Holger Barth

**Affiliations:** 1 Institute of Pharmacology and Toxicology, University of Ulm Medical Center, Albert-Einstein-Allee 11, Ulm 89081, Germany; stefan.carle@uni-ulm.de; 2 Institute of Experimental and Clinical Pharmacology and Toxicology, University of Freiburg, Freiburg 79104, Germany; thorsten.brink@pharmakol.uni-freiburg.de (T.B.); joachim.orth@pharmakol.uni-freiburg.de (J.H.C.O.); klaus.aktories@pharmakol.uni-freiburg.de (K.A.); 3 Centre for Biological Signalling Studies (BIOSS), University of Freiburg, Freiburg 79104, Germany

**Keywords:** *Pasteurella multocida* toxin (PMT), deamidation, G-protein, thioredoxin reductase (TrxR), auranofin

## Abstract

The AB-type protein toxin from *Pasteurella multocida* (PMT) contains a functionally important disulfide bond within its catalytic domain, which must be cleaved in the host cell cytosol to render the catalytic domain of PMT into its active conformation. Here, we found that the reductive potential of the cytosol of target cells, and more specifically, the activity of the thioredoxin reductase (TrxR) is crucial for this process. This was demonstrated by the strong inhibitory effect of the pharmacological TrxR inhibitor auranofin, which inhibited the intoxication of target cells with PMT, as determined by analyzing the PMT-catalyzed deamidation of GTP-binding proteins (G-proteins) in the cytosol of cells. The amount of endogenous substrate levels modified by PMT in cells pretreated with auranofin was reduced compared to cells treated with PMT alone. Auranofin had no inhibitory effect on the activity of the catalytic domain of constitutively active PMT in vitro, demonstrating that auranofin did not directly inhibit PMT activity, but interferes with the mode of action of PMT in cells. In conclusion, the results show that TrxR is crucial for the mode of action of PMT in mammalian cells, and that the drug auranofin can serve as an efficient inhibitor, which might be a starting point for novel therapeutic options against toxin-associated diseases.

## 1. Introduction

*Pasteurella multocida* toxin (PMT) is one of several virulence factors that can be expressed by the gram-negative bacterium *P. multocida*, which is a pathogen for various livestock [1,2,3]. This toxin belongs to the group of AB-type protein toxins, meaning that it is composed of an enzymatically-active A-domain and a receptor-binding/translocation B-domain. It is the causative agent for atrophic rhinitis in pigs, which leads to the destruction of the turbinates within the nasal cavity and the characteristic deformation of the snout [4]. On the molecular level, PMT specifically deamidates certain heterotrimeric G-proteins. Heterotrimeric G-proteins are composed of G_α_, G_β_, and G_γ_ subunits. While G_β_ and G_γ_ are associated to a dimer, the membrane-associated trimer is only present in the inactivated state when G_α_ is in its GDP-bound inactive state. Upon ligand binding to a G-protein coupled receptor, the exchange of GDP to GTP in G_α_ is induced, leading to dissociation of the activated G_α_ from the G_βγ_ dimer and the subsequent activation of effectors and downstream signaling pathways. This leads to varying cellular effects depending on the individual G-protein subtype which is determined by the particular α subunit [5]. Specific substrates of PMTs enzymatic activity are the subtypes G_αq/11_, G_αi1,2,3_, and G_α12/13_, leading to the deamidation of a target glutamine, thereby prohibiting the intrinsic GTPase activity of G_α_, which inhibits the transition of the bound GTP to GDP keeping the G-protein in a permanently active state [6,7,8]. This results in strong mitogenic and anti-apoptotic effects and modulates cell differentiation [7,9,10,11]. Structurally, PMT is a 146 kDa protein containing 1285 amino acids [12]. It is composed of an N-terminal part that is necessary for toxin uptake and translocation and a C-terminal part which is required for enzymatic activity [13,14]. The C-terminal part can be subdivided into three distinct domains: C1, C2, and C3. Domain C3 possesses deamidase activity. C3 also contains a structurally important disulfide bond that needs to be reduced to achieve full enzymatic activity [15]. Like other bacterial AB-type toxins, PMT exploits the endocytic mechanisms of host cells for its cellular uptake. While the receptor for PMT binding is still elusive, the internalization of the toxin is assumed to be accomplished by receptor-mediated endocytosis, followed by endosomal trafficking and acidification, leading to the release of the enzymatically active domain of PMT from acidified endosomes into the cytosol [16,17].

In the present study, we investigated the molecular mechanisms underlying the transport of the enzyme domain of PMT across cell membranes. Cultured human cells were protected from intoxication with PMT in the presence of auranofin. Auranofin—a small molecule gold compound which was originally used as a treatment for rheumatoid arthritis—has been shown to be an effective and specific inhibitor of thioredoxin reductase (TrxR) [18]. Treatment of cells with this compound leads to an alteration of the reductive potential of the cytosol, which efficiently protected cells from intoxication with other bacterial toxins that also contain structurally important disulfide bonds such as *Clostridium (C.) botulinum* neurotoxin (BoNT) and diphtheria toxin (DT) [19,20].

## 2. Results

**Figure 1:**
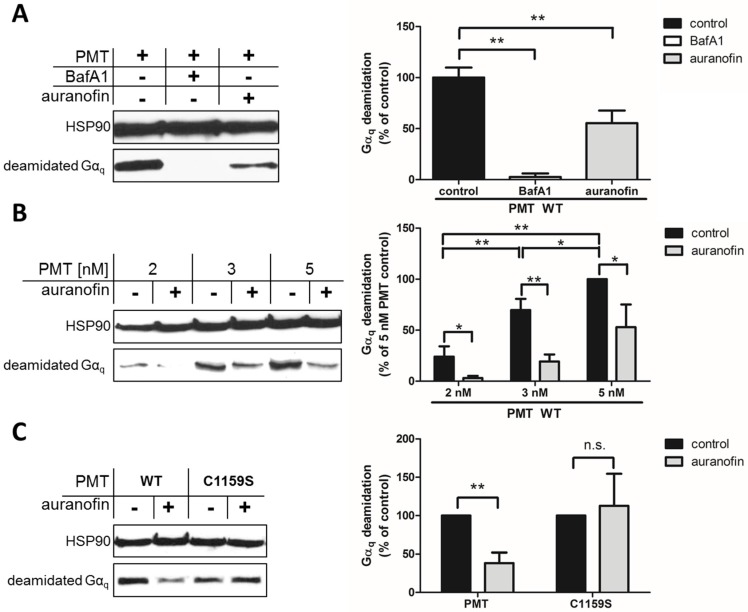
Effect of auranofin on *Pasteurella multocida* toxin (PMT) intoxication of HeLa cells analyzed by Western blotting of deamidated G_αq_ from PMT-treated cells. (**A**) Preincubation with auranofin reduces the deamidation of G_αq_ after treatment of cells with PMT. Cells were pretreated for 30 min at 37 °C in serum-free medium with 1 μM auranofin, 100 nM BafA1, or were left untreated. Then, PMT (5 nM) was added, and cells were further incubated for 3 h. Subsequently, cells were lysed and analyzed by Western blotting with a specific antibody for deamidated G_αq_. Comparable protein loading was confirmed by detecting HSP90 from the lysates. The bar graph shows quantified levels of G_αq_ (normalized to HSP90 loading control and treatment control set as 100%); (**B**) Effect of increasing PMT concentrations on substrate modification levels with or without inhibitor treatment. Bar graph shows quantified levels of G_αq_ (normalized to HSP90 loading control and treatment control of 5 nM PMT set as 100%); (**C**) Effect of auranofin on the intoxication of cells with either wild-type PMT (5 nM) or the mutated activated PMT_C1159S_ (5 nM). Bar graph shows quantified levels of G_αq_ (normalized to HSP90 loading control and treatment control set as 100%). Significance was tested by using the Student’s *t*-test. * *p* < 0.05, ** *p* < 0.01, n.s. = non-significant (*n* = 3).

Since most cell types show no obvious morphological changes after treatment with PMT, the intoxication of cells is determined by analyzing the specific PMT-catalyzed deamidation of G_α_ subunits in the host cell cytosol by Western blotting with a specific antibody against the deamidated form of G_αq_ [8,15]. In this assay, deamidated G_αq_ serves as a marker protein for the PMT-catalyzed modification of G_α_ proteins in cells, but this antibody also detects several other deamidated G_α_ subtypes [8,15]. This approach allows the specific and sensitive detection of the enzyme activity of PMT in cells, because no signal is detected in untreated cells (not shown) or in cells treated with PMT in the presence of bafilomycin A1 (BafA1) (Figure 1A). This well-established inhibitor prevents endosomal acidification, by inhibition of vacuolar-type H^+^-ATPases, and inhibits the translocation of the enzyme domain of PMT into the cytosol, as shown previously [16,17,21].

By using this approach, we first demonstrated that preincubation of HeLa cells with auranofin reduced the PMT-catalyzed deamidation of G_αq_ in PMT-treated cells (Figure 1A). In cells treated with PMT alone, there was a higher amount of deamidated G_αq_, while in cells treated with PMT in the presence of BafA1, no deamidated G_αq_ was detected, indicating that the enzyme domain of PMT did not reach the cytosol. The inhibitory effect of auranofin was also shown by treatment of cells with increasing concentrations of PMT in the absence or presence of auranofin (Figure 1B). The results clearly indicate that less G_αq_ was deamidated in PMT-treated cells in the presence of auranofin, meaning that less active PMT was in the cytosol, suggesting that the TrxR activity of cells is crucial for the mode of action of PMT. However, from this observation, it could not be concluded whether the enzyme activity of PMT was inhibited in auranofin-treated cells or whether less of the enzyme domain of PMT reached the cytosol in cells treated with PMT in the presence of auranofin.

To address this question, we exploited a PMT mutant without the disulfide to investigate the molecular mechanisms underlying the inhibition of PMT intoxication by auranofin in more detail. In wild-type PMT, the peripheral cysteine residue at position 1159 is in close proximity to another cysteine residue at position 1165 in the C3-deamidase domain of PMT, which is assumed to play a major role in the enzymatic activity of PMT. Both cysteine residues form a disulfide bond that needs to be reduced to enable full enzymatic activity [7], but the reducing system in cells that catalyzes this cleavage is not known. By exchange of the peripheral cysteine residue 1159 to a serine residue, no disulfide bond is formed, and PMT_C1159S_ is enzymatically active—even under non-reducing conditions [15]. Therefore, PMT_C1159S_ was tested in direct comparison to wild-type PMT regarding the inhibitory effect of auranofin in a cell intoxication experiment. In the Western blot analysis shown in Figure 1C, there are comparable amounts of deamidated Gα_q_ from cells treated with PMT_C1159S_ independent of whether or not cells were pretreated with auranofin. In contrast, pretreatment of cells with auranofin clearly reduced the amount of deamidated G_αq_ in cells treated with wild-type PMT (Figure 1C). Taken together, this result clearly indicates that the disulfide bond in the enzyme domain of PMT is crucial for the inhibitory effect of auranofin towards the intoxication of cells with PMT. The finding that intoxication of cells by PMT_C1159S_ was not reduced by auranofin indicates that the transport of PMT_C1159S_ into the host cell cytosol was independent of TrxR activity. This suggests that TrxR of host cells might not be crucial for the translocation of PMT from endosomal vesicles into the cytosol, but is involved in the reduction of the disulfide bond in PMT to activate its enzyme domain in the cytosol of living cells.

So far, the results suggest that the inhibitory effect of auranofin is dependent on the inhibition of TrxR, which in turn prevents the reduction of the disulfide bond in the catalytic domain of PMT. To exclude the possibility that auranofin has a direct effect on the enzymatic activity of PMT, a cell-free enzyme activity assay was performed.

**Figure 2:**
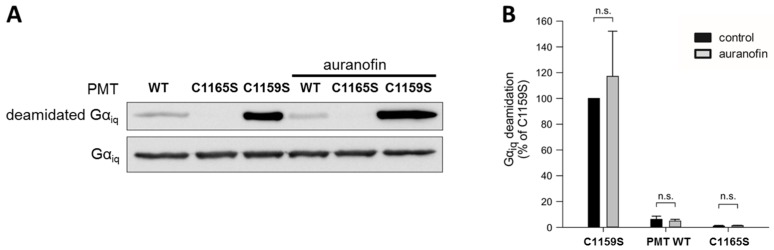
Western blot analysis to detect the enzyme activity of the catalytic domains of wild-type and mutated PMT in a cell-free system. (**A**) Recombinant G_αiq_ was incubated at 16 °C for 20 h with the recombinant C3-deamidase domains of either wild-type (WT) PMT, enzymatically inactive PMT_C1165S_, or PMT_C1159S_ in the presence or absence of 1 μM auranofin. Western blotting was performed to visualize the amounts of deamidated and total G_αiq_; (**B**) Bar graph shows quantified levels of deamidated G_αiq_ (normalized to total G_αiq_ loading control and C1159S control set as 100%). Significance was tested by using the Student’s *t*-test. n.s. = non-significant (*n* = 3).

To this end, we analyzed the in vitro enzyme activity of the catalytic C3-domain of three different PMT proteins: wild-type PMT, PMT_C1165S_ (which is enzymatically inactive), and PMT_C1159S_, which exhibits full enzyme activity independent of any reducing activities due to the loss of the disulfide bond, as described before. Each of these proteins was incubated with chimeric G_αiq_ protein—a recombinantly expressable variant of G_αq_—in the absence and presence of auranofin, and the amount of deamidated G_αq_ was analyzed by Western blotting (Figure 2).

While incubation with the inactive catalytic domain of PMT_C1165S_ did not result in substrate modification, the catalytic domain of wild-type PMT showed some residual enzyme activity, and a strong deamidation was detected in the samples incubated with the catalytic domain of PMT_C1159S_, which does not require disulfide reduction to exhibit its enzyme activity. In each case, however, the presence of auranofin had no obvious effect on the enzyme activity. The result that auranofin did not reduce the enzyme activity of PMT_C1159S_ clearly indicates that this inhibitor did not act directly on the catalytic domain of PMT, but most likely by inhibiting the TrxR in cells.

Taken together, our findings provide strong evidence that during cellular uptake of PMT, the TrxR plays a crucial role in the activation of the catalytic domain of PMT in the host cell cytosol.

## 3. Discussion and Conclusions

Bacterial protein AB-type toxins show a variety of host cell dependencies, and very efficiently exploit cellular mechanisms with a completely different physiological intention, such as receptor-mediated endocytosis for their internalization into cells and protein folding machineries of host cells for the subsequent translocation of their enzymatically active subunits into the cytosol [22,23]. In recent years, a novel host cell dependency came into focus in the context of the cellular uptake of bacterial toxins that contain structurally-relevant disulfide bonds. For the clostridial tetanus and botulinum neurotoxins, Pirazzini and colleagues reported that the reductive conditions of the cytosol are necessary for their full activity [19]. Similarly, in previous work from our group, it was demonstrated that the intracellular membrane transport of the enzyme subunit of diphtheria toxin is effectively inhibited by driving cellular conditions to a more oxidative state [20]. Both studies used auranofin as a specific inhibitor of TrxR in living cells, thereby reducing the potential of the cytosolic disulfide bond reduction. Additionally, all tested toxins have a structurally important disulfide bond connecting their enzymatically active domain which needs to be translocated into the cytosol, with their corresponding delivery domain. Therefore, cytosolic reduction of this bond is a requirement for the toxin to be fully functional.

Here, we show that this necessity is not limited to such inter-domain disulfide bonds, but is also important for the activation of the intra-domain disulfide within the enzymatic domain of PMT. The dependency of reductive conditions for the full activity of wild-type PMT has already been demonstrated before [15], but the relevance for intoxication in a cell-based assay was not given. First trials with auranofin as an inhibitor revealed that there is a defined decrease in amount of modified substrate when cells were pretreated with the compound prior to the application of PMT. Compared to treatment with BafA1, there was no complete inhibition of the enzymatic effect, but an obvious reduction. This could be explained by the presence of alternative reduction pathways [24] and empirically determined dose limitations of auranofin that consider toxic effects on cells. PMT intoxication with increasing amounts of toxin led to increased substrate deamidation in the cells, which could also be inhibited by pretreatment of the cells with auranofin. This indicates that the inhibitory effect cannot just be circumvented by increasing the amount of enzymatically active agent, but is directly dependent on the reductive potential of the cytosol, and thereby on the amount of activated PMT. Additional evidence that auranofin indirectly inhibits activation of PMT via pharmacological modulation of a cellular activation process was given by using PMT_C1159S_, which has no disulfide bond formed, and is therefore in a constitutively active state. While wild-type PMT activity was decreased in the presence of auranofin, no such effect was detected in the PMT_C1159S_ samples, showing that this mutant does not need the reductive potential of the cytosol.

To exclude that the auranofin effect towards PMT-induced cell intoxication is caused by a direct action on the enzymatically active C3-domain of PMT, we analyzed the enzyme activity of different PMT variants with recombinant G_αiq_ in vitro. Under these conditions (without a reducing system, e.g., TrxR), the activity of wild-type PMT was not influenced by auranofin. As expected, the inactive mutant PMT_C1165S_ was without activity, and PMT_C1159S_ exhibited the highest activity because it cannot form an inactive disulfide bond. The finding that auranofin did not alter the activity of any of the PMT variants supports the view that the inhibitor does not directly interact with the enzymatic domain of the toxin.

**Figure 3:**
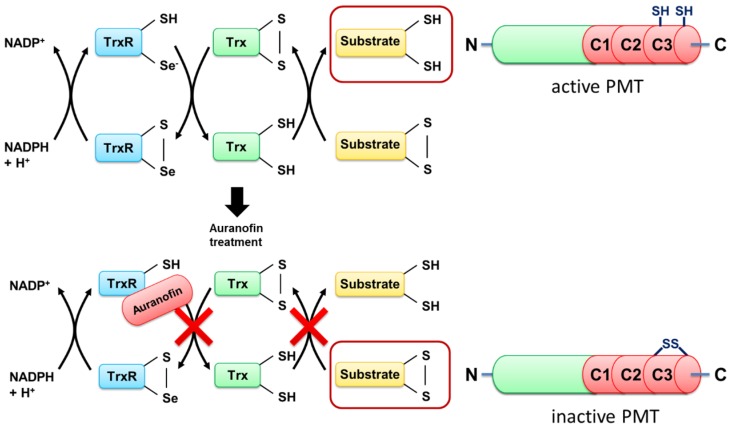
Schematic representation of PMT and the cellular mode of action of auranofin. In the native state, endogenous reduced thioredoxin reductase (TrxR) reduces thioredoxin (Trx), which leads to the reduction of various substrate molecules within the cell. Thereby, also reducing the disulfide bond of the C3-domain of PMT, which leads to its activation. Auranofin binds to the selenocysteine of reduced TrxR, which blocks the downstream reduction of substrate molecules, keeping PMT in its inactive state. Our results suggest that PMT is used as a substrate of the TrxR system, and needs the reducing environment of the cytosol for its cytotoxicity.

Taken together, the results provide evidence that TrxR of host cells is crucial for the activation of the enzyme domain of PMT in the cytosol of mammalian cells, and this process is inhibited by the compound auranofin, a specific pharmacological inhibitor of TrxR in cells (Figure 3).

Moreover, the findings demonstrate that auranofin can serve as an effective pharmacological inhibitor to protect human cells from an intoxication with PMT. This demonstrates that auranofin not only acts against bacterial protein toxins with structurally important inter-domain disulfide bonds [19,20], but also against a toxin that harbors a disulfide bond within its enzymatically active domain. The results not only contribute to a better understanding of the cellular uptake of PMT, but might be a starting point for the development of novel therapeutic strategies against PMT-associated diseases.

In conclusion, the exploitation of the reductive cytosolic conditions of mammalian target cells for toxin activation is likely a feature which is shared between various bacterial toxins that are not closely related to each other. Therefore, auranofin—a licensed drug for treatment of other human diseases—might serve for prevention and/or treatment of diseases which are associated with certain bacterial toxins.

## 4. Materials and Methods

### 4.1. Materials

Chemicals and salts were obtained from Carl Roth (Karlsruhe, Germany) and AppliChem (Darmstadt, Germany). Cell culture media and supplements were obtained from Gibco (Karlsruhe, Germany). Primary α-HSP90 antibody (mouse) and secondary goat-α-mouse-horseradish peroxidase (HRP) antibody were obtained from SantaCruz Biotechnology (Heidelberg, Germany). Secondary goat-α-rat-HRP antibody was obtained Cell Signaling (Danvers, MA, USA). Deamidated G_αq_ specific antibody (rat) was kindly provided by Dr. Y. Horiguchi (Osaka University, Japan).

### 4.2. Cell Culture

HeLa cells were cultivated in Minimum Essential Medium (MEM) supplemented with 10% fetal calf serum (FCS), 1% sodium pyruvate (100 mM), 1% non-essential amino acid solution (NEAA), 1% glutamine (200 mM), and 1% penicillin/streptomycin solution. Standard incubation was at 37 °C in a 5% CO_2_ atmosphere with constant humidity until cells reached near confluency on the dish. Cells were trypsinized and diluted twice a week for continuous cell culture.

### 4.3. PMT and PMT Mutants

PMT, PMT fragments, and PMT mutants were cloned into pCOLDII expression vector system by standard cloning techniques and expressed as *N*-terminal His_6_ fusion constructs [6].

### 4.4. PMT Cell Intoxication Assay and Western Blot Detection of Deamidated G_αq_

For the PMT intoxication assay, 8 × 10^5^ HeLa cells were seeded in a 12-well plate 48 h prior to the assay in 1 mL of serum containing MEM. Cells were then treated with PMT_WT_ or PMT_C1159S_ (concentrations indicated in results) in 1 mL serum-free MEM for 3 h at 37 °C, 5% CO_2_, and constant humidity. Intracellular acting inhibitors (BafA1 and auranofin) were preincubated in 1 mL serum free MEM 30 min prior to the intoxication in corresponding samples, and were also present during the whole incubation time. After intoxication, incubation medium was discarded, and cells were washed twice with PBS-buffer (137 mM NaCl, 2.7 mM KCl, 8 mM Na_2_HPO_4_, 1.8 mM KH_2_PO_4_, pH 7.4). Successively, cells were lysed with 40 μL of hot (95 °C) 2.5-fold Laemmli sample-buffer (150 mM Tris-Cl pH 6.8, 5% SDS (*w*/*v*), 18.75% glycerol, 0.25 mg/mL bromophenol blue) per well. Entire lysate mixtures were then applied to protein gels and subjected to SDS-PAGE with subsequent Western blotting on 0.45 μm nitrocellulose membranes. Unspecific binding sites were then blocked with 5% skimmed milk powder (*w*/*v*; diluted in PBS-T—PBS supplemented with 0.1% Tween 20 (*v*/*v*)) for 1 h at room temperature on a rocking shaker with 25 rpm. Membranes were then split into two parts at the 70 kDa ladder band. The upper part was treated with α-HSP90 antibody (mouse, 1:1000 diluted in PBS-T), and the lower part with α-deamidated G_αq_ antibody (rat, 1:250 diluted in PBS-T). Both membranes were incubated overnight at 4 °C on a rocking shaker (50 rpm). Afterwards, they were treated with corresponding secondary antibodies (either goat-α-mouse-HRP or goat-α-rat-HRP; 1:2500 diluted in PBS-T) for 1 h at room temperature on a rocking shaker. HRP was then visualized using luminescence substrate and X-ray films.

### 4.5. In Vitro Enzyme Activity of PMT

To test for in vitro activity of PMT and its mutants, samples with 20 μL of assay buffer containing recombinant G_iq_ α-subunits (1 μM) were incubated with PMT-C3 deamidase domains (10 nM) of either wild-type PMT, the activated mutant (PMT_C1159S_), or the inactivated mutant (PMT_C1165S_) in the presence of either auranofin (1 μM) or its solvent DMSO (0.02%, control) for 20 h at 16 °C. Samples were then supplemented with 5-fold Laemmli sample buffer and subjected to SDS-PAGE with subsequent Western blotting. For detection of deamidated G_αq_, membranes were treated as described above. As loading control, G_αq_ was detected with G_αq/11_ (C-19) antibody (rabbit, 1:2000 diluted in TBS-T/5% BSA (bovine serum albumin)) and goat-α-rabbit antibody (1:2500 diluted in TBS-T) as secondary antibody.

### 4.6. Reproducibility of the Experiments

Experiments were performed independently at least three times and results from representative experiments are shown in the figures.
